# Best Practices in Designing, Sequencing, and Identifying Random DNA Barcodes

**DOI:** 10.1007/s00239-022-10083-z

**Published:** 2023-01-18

**Authors:** Milo S. Johnson, Sandeep Venkataram, Sergey Kryazhimskiy

**Affiliations:** 1 Department of Integrative Biology, University of California Berkeley, Berkeley, CA 94720, USA; 2 Department of Ecology, Behavior and Evolution, University of California San Diego, La Jolla, CA 92093, USA

## Abstract

Random DNA barcodes are a versatile tool for tracking cell lineages, with applications ranging from development to cancer to evolution. Here, we review and critically evaluate barcode designs as well as methods of barcode sequencing and initial processing of barcode data. We first demonstrate how various barcode design decisions affect data quality and propose a new design that balances all considerations that we are currently aware of. We then discuss various options for the preparation of barcode sequencing libraries, including inline indices and Unique Molecular Identifiers (UMIs). Finally, we test the performance of several established and new bioinformatic pipelines for the extraction of barcodes from raw sequencing reads and for error correction. We find that both alignment and regular expression-based approaches work well for barcode extraction, and that error-correction pipelines designed specifically for barcode data are superior to generic ones. Overall, this review will help researchers to approach their barcoding experiments in a deliberate and systematic way.

## Introduction

Observing how clonal populations of cells change over time is a key to many problems in evolution, development, cancer, and other fields. Until recently, tracking cell lineages was a slow and labor-intensive process (Conklin 1905; Serbedzija et al. 1989; Holland and Varmus 1998; Kretzschmar and Watt 2012; Hsu 2015). Recent advances in genetic engineering and nucleic acid sequencing technologies spurred the development of a new generation of high-throughput lineage tracking methods based on DNA “barcodes” (Blundell and Levy 2014; Woodworth et al. 2017; Kebschull and Zador 2018; Masuyama et al. 2019; Baron and van Oudenaarden 2019; Wagner and Klein 2020; VanHorn and Morris 2021; Dujardin et al. 2021). In these approaches, individual cells are tagged with unique genetic markers called “barcodes.” Many thousands of cell lineages carrying different barcodes can be tracked within a population over multiple generations using high-throughput sequencing. Although barcode lineage tracking (BLT) techniques are fairly nascent, they already found many applications, e.g., for characterizing T-cell recruitment (Schumacher et al. 2010), tracing cellular differentiation over the course of organismal development (McKenna et al. 2016; Frieda et al. 2017; Alemany et al. 2018; Wagner et al. 2018; Weinreb et al. 2020), studying the clonal history of metastasis in cancer (Wagenblast et al. 2015; Bhang et al. 2015; Roh et al. 2018; Umkehrer et al. 2021; Gutierrez et al. 2021; Fennell et al. 2022), screening and characterizing mutant libraries (Giaever et al. 2002; Bell et al. 2014; Wetmore et al. 2015; Li et al. 2019; Johnson et al. 2019; Schubert et al. 2021), identifying the provenance of microbial strains (Qian et al. 2020), and studying evolutionary dynamics (Levy et al. 2015; Cira et al. 2018; Al’Khafaji et al. 2018; Nguyen Ba et al. 2019; Jasinska et al. 2020; Fasanello et al. 2020). With such rapid growth, many methods have been developed for designing, sequencing, and identifying barcodes in the raw sequence data. Multiple labs have independently developed their own BLT procedures without necessarily evaluating pros and cons of other methodologies. Here, we review various existing approaches to BLT experiments and identify some of the best practices for generating and reading barcodes. Some downstream analyses of barcode data, such as estimates of lineage fitness, are discussed in other articles in this special issue (e.g., Limdi and Baym 2022; Li et al. 2023).

BLT studies fall into two modalities (Woodworth et al. 2017; Kebschull and Zador 2018; Baron and van Oudenaarden 2019). *Retrospective* studies, which are typically carried out in the context of development, infer the lineage history of a population of cells based on naturally occurring somatic genetic variation at highly mutable loci, such as microsatellites, which can be viewed as barcodes (e.g., Reizel et al. 2011, 2012). In *prospective* studies, random DNA barcodes are introduced into an organism by the experimentalist to observe future changes. Barcodes diversity is usually generated in vitro, i.e., before the barcodes are integrated into the organism’s genome (e.g., Giaever et al. 2002; van Heijst et al. 2009; Levy et al. 2015; Bhang et al. 2015; Johnson et al. 2019; Ge et al. 2020; Eyler et al. 2020). More recent methods have also been developed that integrate a targeted-mutagenesis module into the organism which then generates barcode diversity at the barcode locus in vivo (e.g., Peikon et al. 2014; McKenna et al. 2016; Frieda et al. 2017; Raj et al. 2018; Spanjaard et al. 2018; Kalhor et al. 2018; Chan et al. 2019). In this review, we discuss DNA barcodes used for prospective lineage tracking, with a specific focus on in vitro barcoding approaches, although some of the discussion will be relevant to other cases as well.

Early prospective lineage tracking studies synthesized and engineered barcodes into individual strains (e.g., different gene-deletion mutants) and then pooled them for the tracking experiment (Giaever et al. 2002; Smith et al. 2009). This approach has the advantage that barcode sequences are known a priori, but it is expensive and labor intensive. Today, barcoded strains are typically generated in bulk by transforming populations of cells with libraries of constructs that contain a diversity of random DNA barcodes, so that barcode sequences of the transformed lineages are initially unknown. The number of distinct cell lineages in such a barcoded population can range from hundreds (Cira et al. 2018; Fasanello et al. 2020) to millions (Bhang et al. 2015; Umkehrer et al. 2021). To track lineages, the population is sampled at one or more timepoints, and the PCR-amplified barcodes are sequenced, typically on the Illumina platform. The relative abundance of each barcode at each timepoint can be estimated from these data, which can then be used for downstream analysis, e.g., estimating mutant enrichment over the course of the experiment.

Researchers who seek to use in vitro-generated barcodes for prospective lineage tracking face a number of choices with respect to barcode design, sequencing, and barcode identification. These include questions regarding barcode length and base composition, strategies for barcode amplification, methods for extracting barcodes from raw sequencing data as well as methods for error correction. Previous studies have implemented a variety of solutions to each of these problems, but we are unaware of any systematic review or comparison of various approaches. Here, we review current practices in barcode design, sequencing and identification, discuss the implications of various choices, and identify current best practices for designing and conducting lineage tracking experiments using DNA barcodes. In the Appendix, we also briefly discuss a related problem of high-throughput genotyping of clones at a barcode locus.

## Barcode Design, Synthesis and Integration

Designing DNA barcodes involves a number of decisions. How long should the barcode locus be? What should be its base composition? Where in the genome will it be integrated? etc. These choices can have various downstream implications, e.g., for the number of lineages that can be tracked, for the fidelity of barcode amplification and sequencing and for the accuracy with which lineage frequencies can be estimated. In this section, we discuss some design considerations for the barcode locus itself (“Structure of the Barcode Locus” section) as well as some practical decisions involved in the construction of a barcoded strain library (“Synthesis and Integration” section).

### Structure of the Barcode Locus

In essence, barcodes are simply random sequences of nucleotides. Most DNA synthesis companies offer an option of including random nucleotide bases into oligonucleotide sequences. Such “barcode” oligos are chemically synthesized and then incorporated into plasmids and/or directly into the genome.

For transposon mutagenesis sequencing (TnSeq) experiments, it is also possible to treat the nucleotides adjacent to the transposon (sometimes called the “edge sequence”) as a “barcode” that identifies the genomic location of each transposon insertion (van Opijnen et al. 2009). However, since sequencing and analyzing the edge sequences are somewhat difficult, researchers often engineer synthetic random barcodes into the transposon, particularly when the same strain library is used in multiple BLT experiments (Wetmore et al. 2015; Johnson et al. 2019). In these studies, only one “difficult” sequencing experiment is required to associate each barcode with its corresponding edge sequence (and, therefore, with its insertion location). Then, in subsequent BLT experiments, one sequences only the random barcodes (Wetmore et al. 2015), which is relatively easy.

In this section, we discuss only the structure of the barcode locus itself and leave out the discussion of other parts of the oligos that may be necessary for engineering and sequencing purposes or other experiment-specific considerations. For example, if restriction enzymes are being used during the cloning process or if it is critical that barcodes are not subject to native restriction endonucleases, one should consider how frequently recognition sites will appear in the barcode design by chance.

**Fig. 1:**
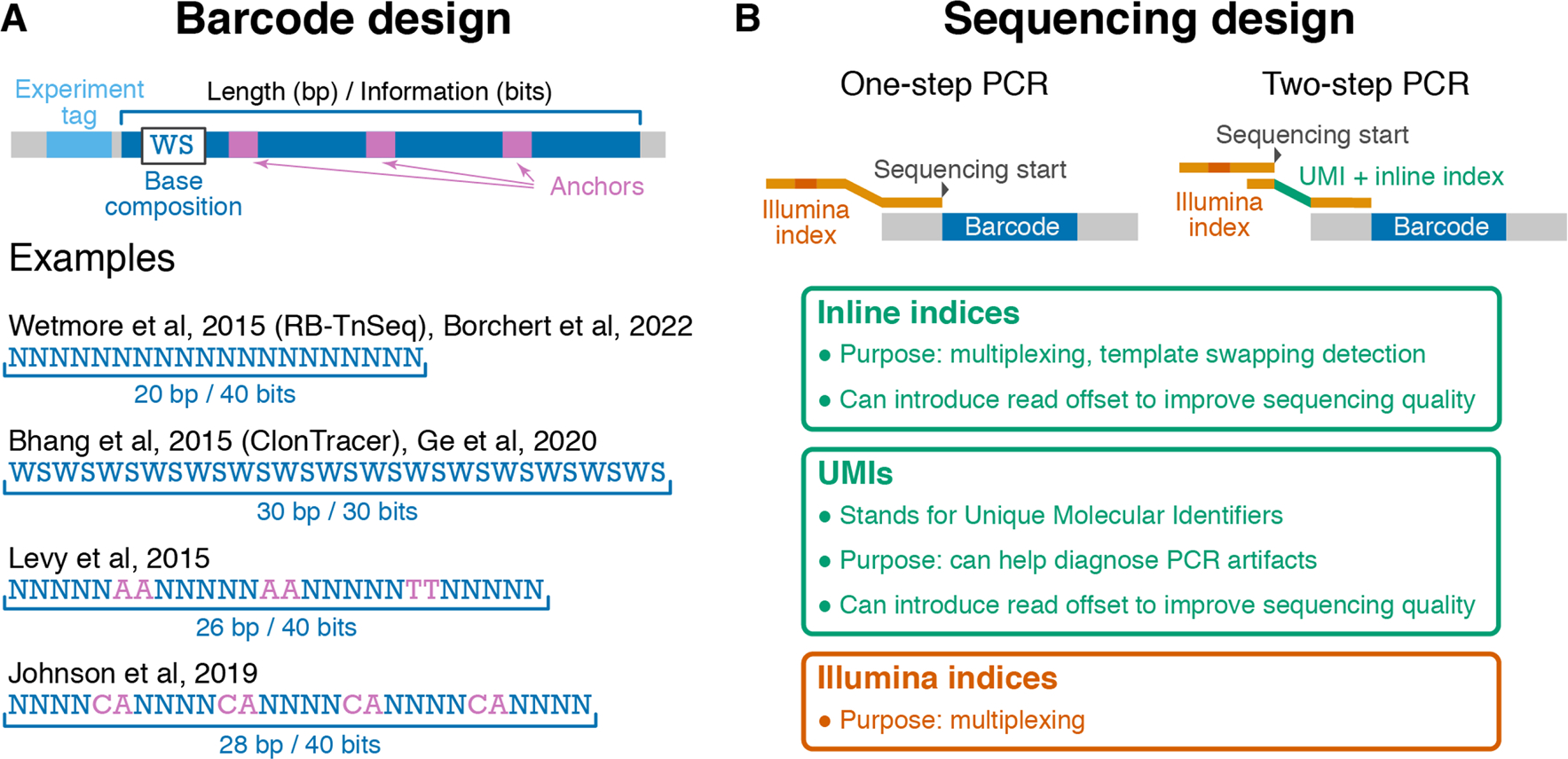
Barcode and sequencing design considerations. **A** Structure of the barcode locus and examples of published barcode designs. “N” represents fully degenerate positions (“A,” “C,” “G,” or “T”), W (“A,” “T”) and S (“G,” “C”) represent partially degenerate positions. **B** Two commonly used barcode amplification strategies, one-step PCR (left) and two-step PCR (right). Key features on the primer sequences are indicated and explained in boxes. The optional experiment tag region on the template DNA is not shown for clarity. Note that in some one-step PCR strategies, inline indices with offsets are included, and sequencing starts at a similar location as in the two-step PCR strategy

The simplest barcodes can be formed by a sequence of random nucleotides, i.e., a sequence of “N”s in the oligo design (see Wetmore et al. 2015 design in Fig. 1A). Other existing barcode designs feature short constant “anchor” sequences that break up “variable” regions (see Levy et al. 2015 and Johnson et al. 2019 designs in Fig. 1A) or consist of alternating random bases that are constrained to be strong (“S,” i.e., G or C) or weak (“W,” i.e., A or T; see Bhang et al. 2015 design in Fig. 1A). Although these designs were motivated by various considerations, such as to balance GC content and reduce PCR amplification biases (Bhang et al. 2015), how well they achieve these goals has been unclear. In fact, we will show below that some designs produce barcodes that are more likely to exhibit extreme GC-content or long repetitive regions (e.g., “AAAAAA ”), which can lead to high error rates and PCR amplification or sequencing efficiency biases. We then discuss the considerations that determine the length of the barcode and describe our new proposed barcode design that we expect to perform better than existing alternatives. We conclude this section with a brief discussion of “pre-multiplexing,” a way of leveraging barcode design to reduce labor and material costs at the library preparation stage.

### Anchors and GC-Content Control

**Fig. 2:**
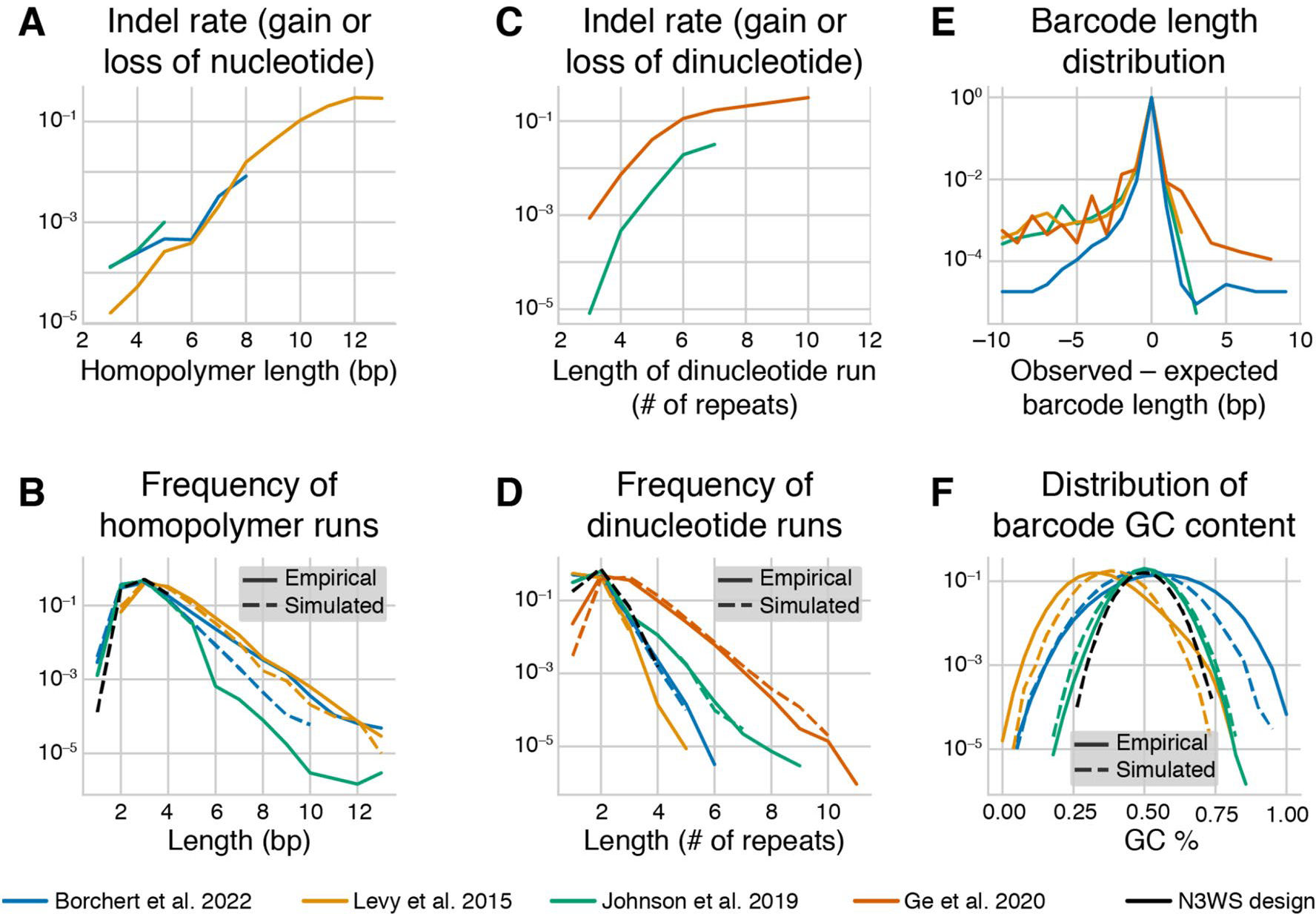
Barcode design features and error rates. (**A**) The total indel error rate in homopolymer runs, estimated from barcode data in the datasets indicated in the legend. Barcode designs are shown in Fig. 1 and Table S1. **(B)** The frequency of homopolymer runs of different lengths in the empirical and simulated datasets of barcodes with different designs (see Methods for details). **(C)** The total indel error rate in dinucleotide runs, estimated from barcode data. **(D)** The frequency of dinucleotide runs of different lengths in the empirical and simulated datasets of barcodes with different designs. **(E)** The empirical distributions of barcode lengths, putatively due to variation in the true length of barcodes (see Methods for details). **(F)** The distributions of GC content in barcodes in the empirical and simulated datasets

The sequence of the barcode matters. To demonstrate this, we reanalyzed data from six barcode sequencing datasets (Table S1). We found that the empirical rate of indel errors that occur during PCR and/or sequencing increases exponentially with homopolymer run length (Fig. 2A) and with dinucleotide run length (Fig. 2C). For runs with more than 10 repeats of a single nucleotide or dinucleotide, up to 30% of reads associated with a barcode have an insertion or deletion in the repetitive sequence. Simulations predict that the prevalence of repetitive DNA sequences varies with the barcode design, and these predictions are quantitatively supported by the data (Fig. 2B, D). Specifically, long homopolymer runs are most common in barcodes with homopolymer anchor sequences (e.g., “AA,” Levy et al. 2015 design, Fig. 1A), and long dinucleotide runs are most common in barcodes with repeating pairs of twofold degenerate bases (“WSWS…”, Bhang et al. 2015 design, Fig. 1A, also used by Ge et al. 2020 and Eyler et al. 2020) or repeated dinucleotide anchors (e.g., “CA,” Johnson et al. 2019 design, Fig. 1A).

We have also observed that a barcode’s GC content can sometimes dramatically bias its representation in the sequencing data (Figure S1, unpublished data). This bias could be driven by GC-content-dependent differences in the PCR amplification (Aird et al. 2011; Benjamini and Speed 2012; Laursen et al. 2017). The magnitude of this bias has a random component (i.e., the bias is stronger in some libraries than in others, see Figure S1), which could stem from uncontrolled variation in the setup of the PCR reaction, purity of the template, etc. These observations also suggest that GC-content-driven biases can be reduced by constraining GC content of all barcodes to a narrow range. Anchors with balanced GC content (e.g., “CA” anchors as in the Johnson et al. 2019 design) can help achieve this goal (albeit at the expense of increasing the frequency of dinucleotide runs), while the “AA” and “TT” anchors used in (Levy et al. 2015) lead to both low GC-content barcodes (Fig. 2F) and a high occurrence of long homopolymer runs (Fig. 2B). A new barcode design we propose and discuss below is an attempt to minimize each of these potential sources of bias and error (Fig. 2B, D, F, black dashed lines).

### Length and Information

The choice of barcode length is dictated by a balance between several factors. On the one hand, barcodes cannot be too long because of current synthesis and sequencing limitations as well as higher costs. Furthermore, longer barcodes, when read by sequencing, will contain statistically more errors than shorter barcodes. On the other hand, length of the barcode locus, together with its structure and base composition, determines the amount of information that the locus can encode, which in turn limits the number of distinct lineages that can be tracked. Specifically, the information content in bits of each barcode position is given by the logarithm with base 2 of the number of alternative nucleotides that can be present at the position. For example, each position where any one of the four nucleotides can be present with equal probabilities encodes log_2_4 = 2 bits of information, positions where only two different nucleotides are admissible encode 1 bit, whereas anchor positions encode 0 bits. The total information I of a barcode locus is given by the sum of information across all of its positions, such that there are at most 2^*I*^ distinct barcode sequences. In a lineage tracking study, each lineage must be tagged with a unique barcode, so that a barcode locus with information I enables tracking of at most $2^{I}$ distinct lineages. Thus, to track K lineages, the barcode locus must have information content that exceeds $I_{min}={log}_{2}K$ bits. A barcode locus that consists of L random nucleotides (the {N}×L design as in Wetmore et al. (2015), see Fig. 1A) has the highest information content of 2L bits among all barcodes of length L. Thus, tracking K lineages requires the barcode of any design to be longer than $L_{min}=1/2{log}_{2}K$ bp.

In practice, barcodes need to have information I that exceeds $I_{min}$ by several bits (and, consequently, of which length exceeds $L_{min}$ by several bp). Recent studies have successfully tracked $K=10^{5}$ to $K=10^{6}$ lineages ($I_{min}$ between 16.6 and 19.9 bits and correspondingly $L_{min}$ between 8.8 and 10 bp) with barcodes with length between 15 and 20 bp and information content between 30 and 40 bits (see Fig. 1A; Levy et al. 2015; Johnson et al. 2019; Jasinska et al. 2020; Ge et al. 2020; Eyler et al. 2020; Borchert et al. 2022).

There are two reasons why I must exceed $I_{min}$. First, since cells acquire barcoded DNA constructs at random during transformation, the barcode library must be diverse enough to ensure that sufficiently many distinct barcodes are transformed into cells. The wider the distribution of barcode frequencies prior to transformation (for a given I), the lower will be the barcode diversity after transformation. To ensure high post-transformation diversity, a typical barcode should be introduced into at most one cell. This occurs whenever

(1) $$Kf_{\max}\ll 1,$$

where K is the number of barcoded cells and $f_{max}$ is the frequency of the most common barcode sequence present in the library (see Methods, “Conditions for Preserving Barcode Diversity During Transformation” section).

If all barcodes are represented in the library equally (so that their frequencies are $2^{-I}$), condition (1) is satisfied whenever $I>I_{min}$. However, in practice, not all barcodes are present in the library at the same frequency. We have estimated the distribution of barcode frequencies right after the transformation in several existing datasets and found that it is closer to exponential or heavy tailed (Figure S2). When the distribution is wide, $f_{max}>2^{-I}$, and it is advisable to design barcodes with information content exceeding $I_{min}$ by at least a few bits to preserve post-transformation diversity. For example, if the distribution of barcode frequencies in the library is exponential, then we expect.

$f_{max}=2^{-I}(\gamma +I\;ln2)$, where γ≈0.577 is the Euler-Mascheroni constant (Methods, “Conditions for Preserving Barcode Diversity During Transformation” section). Thus, to satisfy criterion (1), I should exceed $I_{min}$ by at least 6 bits whenever the desired information content is between 30 and 60 bits. This criterion has been satisfied in all the datasets that we reanalyzed.

The second reason to increase *I* further is that barcode sequences cannot be amplified or read with perfect accuracy. While errors are inevitable, good barcode designs account for error statistics and enable researchers to correct at least some of them. Sequencing errors can be accounted for most easily. On the Illumina platform, the error rate is estimated to be ≤ 0.4% per sequenced nucleotide (Stoler and Nekrutenko 2021), such that up to 7.7% of reads of a 20-bp (40 bit) barcode are expected to contain at least one error and up to 0.3% are expected to contain two or more errors. Good barcode designs ensure that the true barcode sequence can be correctly inferred despite these errors. All error-correction methods rely on the premise that true barcode sequences are sufficiently sparse in the sequence space, so that they all differ from each other at least at 2, or, better yet, at 4 positions (see “Identifying Barcodes in Sequencing Data” section).

**Fig. 3:**
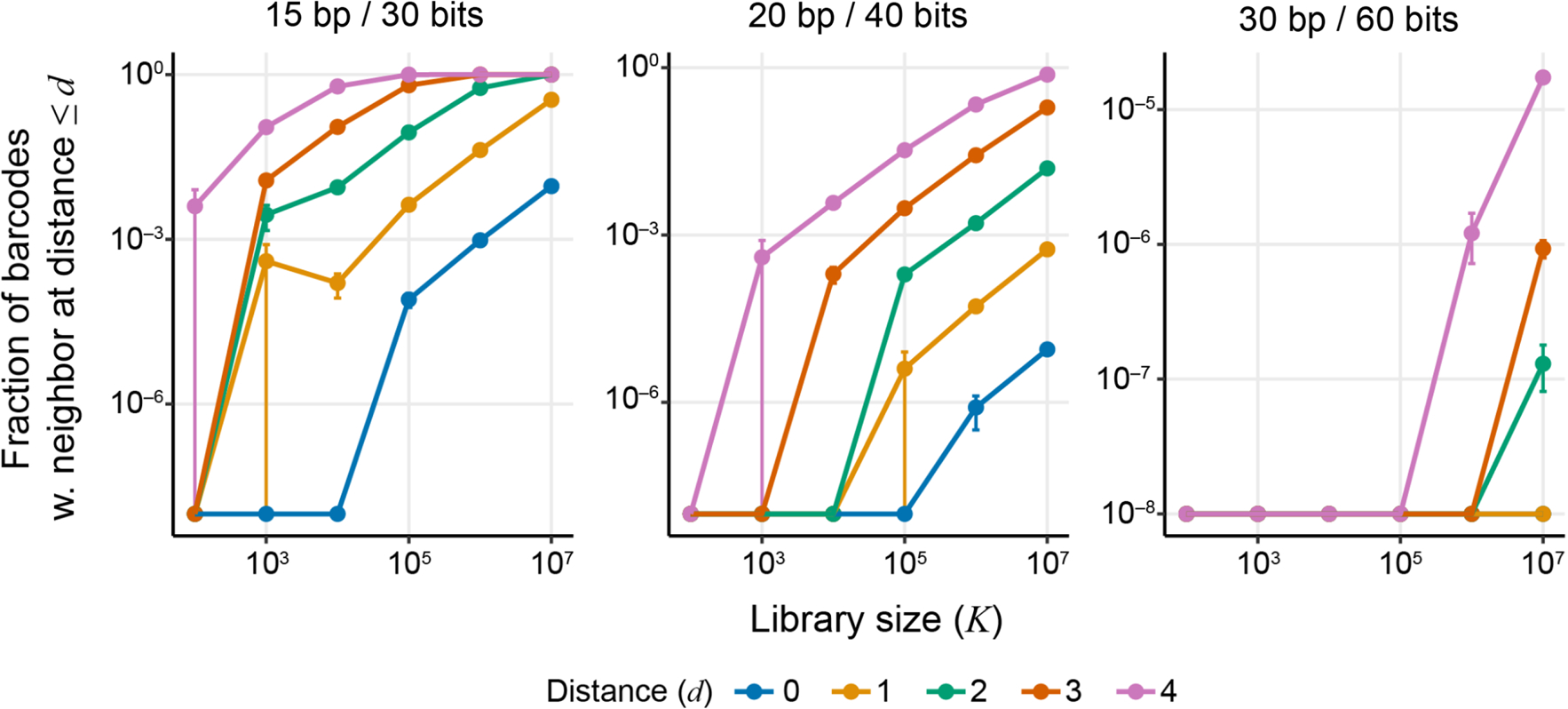
Fraction of barcodes with at least one other barcode within a certain Hamming-distance radius, as a function of library size. Lines correspond to different Hamming-distance radii *d*, as shown in the legend. Panels show barcodes with different lengths and information content. For each library size *K*, five replicate libraries of fully degenerate barcode sequences were simulated and the resulting fractions were averaged over the replicates. Error bars show ± 1 standard error of the mean

To evaluate the error-correction capacity of a given barcode design when tracking K lineages, it is useful to calculate the fraction of K random barcodes that have a nearest neighbor barcode within Hamming distance d. This quantity can be estimated analytically, using techniques from coding theory (Lamberger et al. 2012), or with simulations. Our simulations (see Methods, “Distribution of Hamming Distances Between Barcodes” section) show that this fraction increases rapidly with K (Fig. 3), such that if barcodes of length 15 bp are used to track $K=10^{5}$ lineages, about 8.8% of them have another barcode at Hamming distance 2 or less, which can complicate or compromise our ability to correct many sequencing errors. However, increasing barcode length to 30 bp enables one to track $K\sim 10^{7}$ lineages while maintaining the capacity to correct sequencing errors since only about 0.002% of barcodes have a nearest neighbor within Hamming distance 4 (Fig. 3).

### New Barcode Sequence Design

The considerations discussed above place conflicting demands on barcode design. High information content is most easily achieved by using fully random nucleotides, but such barcodes have problems with GC content and homopolymer runs (Fig. 2). At the same time, full control of the GC content is achieved at a great reduction of information or expansion of length (see Fig. 1A) and can still have problems with dinucleotide runs (Fig. 2). Thus, we propose a new barcode design that achieves a reasonable balance between all these demands. We propose interspersing twofold degenerate “WS” nucleotides between every three fourfold degenerate nucleotides to generate a 38 bp barcode design which we refer to as “N3WS”: “NNNWSNNNWSNNNWSNNNWSNNNWSNNNWSNNNWSNNN.” The two-fold degenerate “WS” bases help control the GC content and limit the length of mononucleotide runs, the fourfold degenerate bases increase the information content and reduce the length of dinucleotide runs. These dinucleotide runs, which can lead to high error rates (Fig. 2C), are more prevalent in alternate designs with 2 or 4 fully degenerate bases between the anchors rather than 3 (Figure S3), though in some cases the added benefit of more information in a shorter barcode may still make a N4WS design preferable.

The N3WS design has 62 bits of information, a guaranteed GC content between 28 and 72%, and maximum homopolymer/dinucleotide run lengths of 4 (Fig. 2B, D, F, black dashed lines). We note however that this barcode design may not be compatible with 50 bp single-end reads, depending on the length of the pre-barcode region (discussed below). If using shorter sequencing reads is a priority, researchers could reduce barcode length to, say, 28 bp (46 bits), and/or consider using a N4WS design.

### Pre-Multiplexing

It is often desirable to sequence barcodes from multiple BLT experiments on one Illumina lane. The standard solution to this problem is to use Illumina indices during library preparation (Fig. 1B and “Barcode Sequencing” section). However, this approach requires that the sequencing library is prepared for every sample individually. It is possible to reduce this labor and material costs by “pre-multiplexing” different BLT experiments.

One pre-multiplexing strategy is to add a short sequence—referred to as the “experiment tag”—next to the barcode (Fig. 1A) and to construct barcoded strain libraries for different BLT experiments with different experiment tags (Boyer et al. 2021). Another strategy is to create multiple plasmid libraries (see “Synthesis and Integration” section) with non-overlapping sets of barcode sequences (Johnson et al. 2019). Of course, these plasmid libraries must be sequenced to determine which barcodes belong to each set. The second strategy can be implemented easily only if the number of tracked lineages is much smaller than the diversity of the library of chemically synthesized barcode oligos.

With either strategy, pre-multiplexed samples can be pooled together prior to DNA extraction and library preparation. The identity of the BLT experiment can then be inferred from the sequence of the “experiment tag” (first strategy) or the barcode itself (second strategy). In addition to or instead of increasing throughput, pre-multiplexing can be used redundantly with standard Illumina multiplexing to avoid potential misidentification of reads due to template switching, index hopping, or primer cross-contamination (see “Barcode Sequencing” section and Johnson et al. (2019)).

### Synthesis and Integration

Once the barcode construct has been designed, the oligonucleotides carrying the barcodes must be synthesized and engineered into the organism. While an in-depth discussion of various engineering methods involved in the barcoding process is beyond the scope of this paper, we outline here the basic steps and then discuss some considerations related to barcode construct synthesis and to the choice of the locus into which barcodes are integrated.

### Overview of the Barcoding Process

The barcoding process usually begins with the synthesis of oligonucleotides carrying the barcode sequences. Such an oligo library is then typically used to generate a library of larger DNA constructs that are ready to be transformed into the organism of interest. These constructs are typically integrated into a plasmid backbone and transformed into *Escherichia coli* for long-term storage. Before each application, plasmids are harvested and transformed into the target organism, either directly (Levy et al. 2015) or after another manipulation step, such as backbone digestion (Jasinska et al. 2020) or lentivirus generation (McKenna et al. 2016). Sometimes, barcodes are integrated into the organism’s genome using high-efficiency recombinase systems, such as transposon-based systems like Tn7 (Jasinska et al. 2020), Cre-Lox (Levy et al. 2015), or CRISPR-Cas9 (Zhu et al. 2019).

It is important to keep in mind that the construction of barcoded strain libraries involves multiple sampling steps, each of which inherently reduces barcode diversity. It is critical to ensure that sample sizes at each step are large enough that the diversity of the barcoded strains is sufficient for the purposes of the BLT experiment.

Another important consideration is that any sufficiently large population harbors beneficial genetic variation, even prior to the barcoding step. As the population grows after the barcoding step, natural selection will elevate the frequencies of these variants, which will lead to unwanted variation in barcode frequencies. Thus, it is advisable to limit the number of generations of growth of the barcoded library, both in *E. coli* and in the target organism, prior to the beginning of the BLT experiment. This is especially important when the introduction of barcoded constructs itself is expected to cause fitness differences between lineages, e.g., when barcodes are associated with transposon insertion mutations. In cases where barcode diversity prior to the BLT experiment is in question, it may be useful to sequence the plasmid library before using it for the transformation of the target organism.

### Synthesis

In vitro barcodes are typically generated using chemical oligonucleotide synthesis, which can result in errors in the length of the barcode as well as its sequence. We estimated the distributions of barcode length in our datasets (see Methods, “Measuring Variation in Synthesized Barcode Length” section) and found that barcodes of abnormal length are indeed present at appreciable frequencies (Fig. 2E). Filges et al. (2021) quantified the error rate of synthesized oligonucleotides from multiple manufacturers and various purification methods, and found that IDT Ultramer and Eurofins PAGE oligonucleotides had similarly high purity (~ 98.4% full-length molecules). Oligonucleotides without any purification (“de-salted”) can result in as low as 86% full-length molecules, and should, thus, be avoided (Filges et al. 2021). In our experience with IDT, ordering “custom/hand mixed” random nucleotides provided a more even frequency distribution than “machine mixed” nucleotides (see https://www.idtdna.com/pages/products/custom-dna-rna/mixed-bases).

### Integration Locus

In some BLT studies, barcodes are integrated into different, sometimes random, genomic locations in different lineages (Giaever et al. 2002; Wetmore et al. 2015; Johnson et al. 2019). But in many others, researchers wish to integrate a barcode into one specific locus, in which case they need to decide what this locus would be. The first decision is whether the barcode will be maintained on the chromosome (Levy et al. 2015; Jasinska et al. 2020) or on an extrachromosomal plasmid (Cira et al. 2018). While the latter strategy is easier to implement, barcodes maintained on plasmids may be less stable (i.e., they can be lost), depending on the organism, growth environment, and the type of plasmid (Friehs 2004; Shao et al. 2021).

The second question is to identify the specific locus for barcode integration. Some considerations that will bear on this decision are study specific, e.g., whether the barcode needs to be expressed (Wagner et al. 2018). Others are more general, such as the aforementioned stability requirement, i.e., the requirement that lineages maintain their barcodes over the course of the experiment. For this purpose, one should avoid barcode integration into recombination hotspots or into loci adjacent to mobile genetic elements. While in our experience, genomic integration of barcodes tends to be stable in most genomic locations, stability can be further enhanced by integrating the barcode in the immediate proximity of an essential gene, such as next to an antibiotic resistance marker (Giaever et al. 2002) or in an intron of an essential gene (Levy et al. 2015).

Another general consideration is that the presence of the barcode should minimally perturb cellular function. For example, in many evolutionary studies, barcodes should ideally have no effect on the organism’s fitness, in which case pseudogenes or genes whose disruption is known to have no effect on fitness in the study environment are good candidates for integration. It is important to keep in mind that neutrality of a locus in one environment does not guarantee its neutrality in other environments.

## Barcode Sequencing

Once a lineage tracking experiment is complete and samples are collected, the next step is to characterize lineage diversity in these samples by sequencing them at the barcode locus. Since the number of barcodes per sample is often very large and their relative abundances can vary by multiple orders of magnitude, sequencing must be done to a substantial depth, often $≳10^{6}$ reads per sample. Our discussion here focuses on the Illumina platform where such depths can currently be achieved at a relatively low cost.

Barcode amplification and sequencing begin with DNA extraction, usually with standard organism-specific methods. Then, PCR is used to simultaneously amplify the barcode locus and attach Illumina adapters necessary to create sequencing-ready DNA fragments. Both the sequencing-library preparation and the sequencing process itself introduce errors into the barcode sequence, which creates difficulties in identifying barcodes in the data and increases noise in the estimates of their frequencies. However, clever PCR designs can help reduce and correct some of these errors, as well as reduce labor and sequencing costs. In particular, we discuss the benefits and pitfalls of using one- versus two-step PCR setups, Unique Molecular Identifiers (UMIs), inline indices, and a few other factors (see Fig. 1B).

### One- and Two-Step PCR Setups

The simplest way to generate sequencing-ready barcode amplicons from a sample’s genomic DNA is to PCR-amplify the barcode locus using primers that contain standard Illumina adapter components, including Illumina multiplexing indices, the sequencing priming site, etc. We refer to this simplest approach as the “one-step” PCR setup (Fig. 1B). A slightly more complex alternative is the “two-step” PCR setup (Fig. 1B). Here, the first PCR is typically carried out for a small number of cycles (2–10). Its purpose is to attach “overhangs” to template molecules. These overhangs contain useful components, such as inline indices, UMIs, and read offsets, which we discuss in detail below, as well as a “universal” priming site for the standard Illumina primers used in the second PCR. The second PCR is typically carried out for a larger number of cycles (12–25) and results in sequencing-ready fragments.

Both setups have some advantages and disadvantages. A major advantage of the two-step PCR setup is that inline indices can greatly expand multiplexing capacity, which not only increases throughput but can also improve data quality (see below). This advantage is traded off against an additional bottleneck in the two-step PCR setup because a fraction of the original template molecules do not receive overhangs (which are necessary for the second PCR) and a fraction of molecules with overhangs are lost during the cleanup after the first PCR. The advantage of the one-step setup is that it avoids this bottleneck, potentially reducing noise, and in general, involves a bit less hands-on work. On the other hand, one-step setup requires (somewhat expensive) long non-standard primers and, most importantly, lacks the multiplexing capacity endowed by inline indices.

Regardless of which setup is chosen, it is critical to keep in mind that amplification is a sampling step and, therefore, introduces measurement noise. To minimize this noise and preserve barcode diversity, the amount of template DNA must be sufficiently large. Specifically, we suggest that the number of templates should greatly exceed the eventual number of sequencing reads (see an extended discussion of this so-called “read-limited” regime in the section Unique Molecular Identifiers below). This may require genomic DNA in μg range with several parallel PCR reactions.

### Inline Indices

A major advantage of a two-step PCR setup is that the inline indices added during the first PCR step greatly expand the multiplexing capacity enabled by standard Illumina indices (Fig. 1B). Like the Illumina indices, inline indices are predefined sequences that encode sample information. For example, each replicate of a BLT experiment can be tagged with its own inline index during the first PCR step. In this setup, sample information can be encoded by a combination of four indices (two Illumina and two inline). In principle, samples tagged with different inline indices during the first PCR can be pooled together for the second PCR, although we do not recommend this practice due to the possibility of template-switching events (Kinsler et al. 2022).

Expanded multiplexing capacity allows for redundant sample encoding whereby all samples are distinguished from each other by at least two indices, e.g., one inline index and one Illumina index. One redundant design that we found particularly useful is where each 5’ inline index is associated with a unique 3’ Illumina index and each 3’ inline index is associated with a unique 5’ Illumina index. Such redundancy can be used to effectively detect primer cross-contamination, “index hopping,” and template-switching events that can occur during library preparation or on the Illumina flow cell (Illumina 2017; Guenay-Greunke et al. 2021; Kinsler et al. 2022). These processes generate chimeric sequences, which introduce demultiplexing errors that in turn translate into errors in lineage frequency estimates. In the aforementioned design, most such events (those that occur in the bulk of the fragment, between the inline indices) generate “inadmissible” index combinations that can be easily identified and discarded. Using this approach, we found that ∼5% of reads had inadmissible index combinations (Venkataram et al. 2022), but others have reported rates of up to 43% (Kinsler et al. 2022). Note that, while it is possible to include inline indices in the one-step PCR setup, their utility would be limited. They cannot expand the multiplexing capacity but can help detect some index hopping events (those that occur between the Illumina index and the inline index that are on the same primer). The rate of index hopping is much higher on “patterned flow cell” Illumina machines, so we also recommend using a non-patterned flow cell machine for barcode sequencing whenever possible (Illumina 2017; Guenay-Greunke et al. 2021; Kinsler et al. 2022).

### Unique Molecular Identifiers (UMIs)

The process of preparing a sequencing library introduces a number of potential errors that may influence the quality of BLT data. In particular, if the number of template molecules that are being amplified by PCR is small, data will be noisy despite high read depth. In addition, sequence-specific biases may arise during PCR (i.e., some barcodes may be amplified more efficiently than others) which can lead to systematically inaccurate frequency estimates (Thielecke et al. 2017). Finally, “jackpot” errors that occur in the first few rounds of PCR can be overrepresented in the final sequencing pool. The two-step PCR setup allows researchers to employ Unique Molecular Identifiers, or UMIs, which can help diagnose these issues. UMIs are random sequences, typically 6 to 10 bp long, present on the first-step PCR primers (Fig. 1B), such that each molecule that serves as a template in the second-step PCR is tagged with one UMI. Once the final DNA fragment is sequenced, the UMI appears at the start of each read and can be used to determine whether multiple reads with the same barcode sequence derive from the same template molecule (Fu et al. 2011; Kivioja et al. 2011).

Although many BLT studies have used UMIs, few have clearly articulated what kinds of insight can and cannot be gained from them. UMI-tagged barcode data allow us to calculate two numbers for each barcode, both of which depend on various experimental parameters, such as the quantity of input DNA and the total read depth: (i) the total number of reads containing the barcode and (ii) the number of unique barcode-UMI combinations among these reads. By dividing the latter by the former and subtracting this ratio from 1, we can obtain the fraction of “UMI duplicates,” i.e., the fraction of redundant reads derived from the same template molecule. To understand how the fraction of UMI duplicates can help diagnose potential PCR problems, consider two extreme cases of the distribution of UMI duplicates across barcodes.

At one extreme, the fraction of UMI duplicates is close to 1 for most barcodes, which means that the same barcode is associated with the same UMI on many reads. In other words, the number of sequenced fragments greatly exceeds the number of original template molecules, so that most reads derive from a small number of templates. We refer to this regime as “template-limited.” At the other extreme, the fraction of UMI duplicates is close to zero for most barcodes, which indicates that UMI duplicates are rare, i.e., almost every read contains a unique barcode-UMI combination. In other words, the number of original template molecules greatly exceeds the number of sequenced fragments, so that most templates are sequenced on at most one fragment. We refer to this regime as “read-limited.”

These regimes differ in two respects. First, given the same total sequencing depth, estimates of lineage frequencies will be noisier in the template-limited regime than in the read-limited regime simply because fewer molecules are being counted. In this sense, the read-limited regime is more cost effective. Second, in the read-limited regime, UMIs provide little information about sequence-specific amplification biases because all templates that are represented in the sequencing data are represented equally (once) and it is unknown which templates are not represented. In contrast, sequence-specific amplification biases (if they exist) can be in principle detected in the template-limited regime because different template molecules may be represented by different numbers of reads. Such biases can also be to some extent corrected by removing UMI duplicates, i.e., by counting unique barcode-UMI combinations rather than counting all reads carrying each barcode. However, the extent to which such biases can be corrected using this simple procedure strongly depends on the fraction of UMI duplicates in the data. In fact, our simulations show that the power to correct biases grows slowly with the fraction of UMI duplicates (Figure S4). For example, if each template molecule is sequenced on average twice, UMI duplicates comprise 50% of reads, but discarding all of them corrects only 40–70% of the underlying PCR biases.

Even if the biases cannot be corrected fully, removing UMI duplicates will in principle improve the estimation of lineage frequencies, in any sequencing regime. However, before removing UMI duplicates, researchers must ensure that the same UMI sequence is unlikely to associate with two distinct template molecules carrying the same barcode just by chance. This undesired event can happen if the UMI diversity is low. For example, if the UMI is 6 bp long, there are only $4^{6}\approx 10^{3}$ distinct UMIs available during the first PCR. If 10^4^ distinct template molecules with a certain barcode are eventually sequenced, each UMI will on average associate with 10 different templates. Removing UMI duplicates in this case would erroneously reduce the abundance of this barcode by a factor of 10. Thus, we recommend removing UMI duplicates only if the number of possible UMI sequences is several orders of magnitude larger than the highest barcode read count. In our own datasets (Johnson et al. 2019; Venkataram et al. 2022), we have not observed any meaningful changes in barcode frequencies when UMI duplicates are removed.

Finally, UMIs can in principle help detect and eliminate “jackpot” PCR errors. However, such errors do not represent a particularly severe problem, at least as long as sequencing is done in the read-limited regime. Consider an error that occurs in the first PCR cycle. The resulting erroneous barcode is present in one copy, which corresponds to a frequency 1/*T* where *T* is the number of distinct template molecules that will eventually be sequenced. This frequency is very low as long as *T* is very large, i.e., in the read-limited regime. Since all these template molecules are amplified roughly equally during the subsequent PCR cycles (module PCR biases, discussed above), the erroneous barcode will be present at the same low frequency in the read data. Therefore, error-correction methods discussed in “Identifying Barcodes in Sequencing Data” section should be able to handle jackpot PCR errors along with sequencing errors, and UMIs are not required just for this purpose.

In summary, the distribution of UMI duplicates can help us determine the sequencing regime. Sequencing in the read-limited regime will produce data that may contain unobserved PCR biases which can distort barcode frequencies. Sequencing in the template-limited regime will produce noisy data that will still contain biases, unless most of the reads are discarded. Thus, the read-limited regime is preferable in practice because of its cost-effectiveness, and most BLT studies have been done in this regime (Levy et al. 2015; Johnson et al. 2019). It appears more prudent to reduce sequence-specific amplification biases (e.g., GC-content bias) with careful barcode design (see “Structure of the Barcode Locus” section). Thus, in our opinion, if a two-step PCR is required for multiplexing or other practical reasons, it is easy and beneficial to have UMIs on the first-step primers, but we see no fundamental issues with single-step PCR setups without UMIs.

### Read Offsets

Every sequencing-ready fragment must contain a priming site for an Illumina sequencing primer, but there is some flexibility in its location. The standard location is downstream of the Illumina index and upstream of the inline index/UMI region (two-step PCR in Fig. 1B). This location implies that sequencing commences in a region that could have low nucleotide diversity in the sequencing library. Low diversity, particularly at the beginning of a read, can substantially reduce base-call accuracy on the Illumina platform (Illumina 2022). This problem is usually remedied with standard methods, such as spike-in of PhiX or by sequencing a barcode library together with a genomic library on the same lane. A barcode PCR design feature referred to as “Read offsets” can be used in conjunction with these methods to further increase nucleotide diversity at the beginning of barcode reads (Bendixsen et al. 2020). The idea is simply to design a set of first-step PCR primers where either the inline indices or the UMIs have variable length. Such variation creates “read offsets” in the downstream regions of otherwise low diversity (e.g., between the inline index and the barcode), so that fragments with different offsets are read by the sequencer asynchronously, which increases base diversity.

As an alternative, some researchers have designed the barcode locus so that sequencing begins directly at the barcode (Jasinska et al. 2020; Ge et al. 2020; Eyler et al. 2020), which largely avoids the aforementioned base diversity issues. On the other hand, it precludes the use of inline indices for multiplexing and UMIs for estimating library preparation bottlenecks.

### Other Considerations

In our experience, the quality of barcode sequencing data can vary depending on several factors, such as the type of polymerase, the PCR purification and size-selection method. We found that high-fidelity polymerases, especially during the first PCR step, consistently produce better quality data. We also found that bead-based size selection or standard gel extraction works reliably better than strict E-gel-based (Thermo Fisher) size selection. While these simple general practices improve data quality, some biases remain and require more sophisticated approaches, such as those discussed above (see “Structure of the Barcode Locus” section).

During the barcode-edge sequence association step in TnSeq experiments (see “Barcode Design, Synthesis and Integration” section), both chimeric PCR reads and a lack of diversity in the barcode library can lead to reads with identical barcodes but different edge sequences. Therefore, it is important to use a highly diverse barcode library and to computationally screen out chimeric barcode associations (Wetmore et al. 2015).

## Identifying Barcodes in Sequencing Data

Once the sequencing data have been obtained and de-multiplexed, the final technical step is to extract barcodes from sequencing reads and estimate the relative abundances of the lineages.

### Barcode extraction

Extracting barcodes from the sequencing reads may appear as a trivial problem at the first glance, given that the structure of the read is known by design. However, the challenge is that not all reads have identical structure, due to read offsets (see “Read Offsets” section), due to the variability in barcode length that arises during synthesis and due to errors that arise during sequencing-library preparation and sequencing itself. These challenges can be solved using either regular expressions (“regex,” e.g., Levy et al. 2015; Johnson et al. 2019; Chochinov and Nguyen Ba 2022) or sequence alignment (e.g., Jasinska et al. 2020; Venkataram et al. 2022). The former scans each read for certain user-specified patterns of characters, whereas the latter uses sequence alignments to find the locations of constant regions (sequence regions shared by all fragments) flanking the barcode before extracting the barcode sequence between those regions.

We applied both of these approaches to six barcode sequencing datasets (Table S1) to test their speed and relative accuracy (see Methods, “Comparison of Barcode Extraction Methods” section). To compare the two methods, we looked at the first 100,000 reads of each dataset and directly compared extracted barcodes. We found that both methods successfully extracted barcodes from 94 to 98% of reads, with the vast majority of the remaining reads excluded due to low quality scores (Table S2). Excluding reads in which both methods did not extract a barcode (again usually based on low quality scores), the two methods extracted the same barcode in 97.5–99.5% of reads (Table S2). The most common exceptions to this overarching concordance are cases where barcodes have abnormal length. Such barcodes were correctly extracted by the alignment method but were not extracted or extracted incorrectly by our regex method, which only allows barcodes to vary in length by at most 2 base pairs. However, more lenient regular expressions can be developed to allow for more barcode length variation. Indeed, we used regular expressions with no length constraints to examine the distributions of barcode length in our datasets (Fig. 2E). Finally, in very rare cases, both methods extracted incorrect barcode sequences, which happened usually due to misidentification of the constant regions flanking the barcodes.

In our hands, the regex approach ran 5 to 10 times faster than alignment, processing ∼140 million reads in ∼2 h using a basic cloud machine from Deepnote. Given the speed of the regex approach, we believe it will be the method of choice for most applications despite a minor loss of accuracy. When using any method, researchers should pay attention to the fraction of reads without an extracted barcode. This fraction exceeding a few percent indicates a potential problem with sequencing quality, misspecification of parameters of the extraction method (see Methods, “Comparison of Barcode Extraction Methods” section, for parameters we used), or data (e.g., high abundance of abnormal barcodes).

### Error correction

Even with the best practices suggested above, there will be cases when the extracted barcode sequence differs from the sequence of its template molecule. The naive approach is to simply ignore these errors. However, it would come with a substantial data waste (and hence, reduced accuracy of lineage frequency estimates). Assuming a per-base error rate of 0.4% (Stoler and Nekrutenko 2021), 7.7% of sequenced barcodes of length 20 bp contain at least one sequencing error; this fraction is 11% for 30-bp barcodes and 15% for 40-bp barcodes. Moreover, some errors may be sequence-specific (see “Structure of the Barcode Locus” section), such that the naive approach may produce biased lineage frequency estimates. Fortunately, a number of error-correction techniques are available (e.g., Li and Godzik 2006; Edgar 2010, 2016; Ghodsi et al. 2011; James et al. 2018; Wei et al. 2021; Dasari and Bhukya 2022; Millán Arias et al. 2022), some of which were developed specifically for barcode data (e.g., Zorita et al. 2015; Zhao et al. 2018; Tavakolian et al. 2022).

All these methods rely on a few assumptions. True barcodes must be sufficiently sparse in the sequence space, errors must be relatively infrequent, and an erroneous barcode sequence must be more similar to its “parent” barcode than to any other true barcode. With good barcode design and careful sequencing-library preparation, these assumptions are usually met. Then, error correction can be achieved by clustering sequenced barcodes according to a sensible similarity metric, such as Hamming or Levenshtein distance. The primary challenge is computational: BLT data often contain tens or hundreds of millions of reads, and calculating pairwise distances between all of them is not feasible. Clever algorithms that limit the number of comparisons are, thus, key to computational efficiency.

We selected six error-correction software, two developed for generic sequence data, DNAClust (Ghodsi et al. 2011) and CD-Hit (Li and Godzik 2006), and four developed specifically for barcode data, Bartender (Zhao et al. 2018), Starcode (Zorita et al. 2015), Shepherd (Tavakolian et al. 2022), and “Deletion-Correct,” a modified version of the algorithm used in (Johnson et al. 2019). We tested their accuracy by performing error correction on a dataset of simulated barcode reads with realistic errors (Methods, “Comparison of Error Correction Methods” section).

**Fig. 4:**
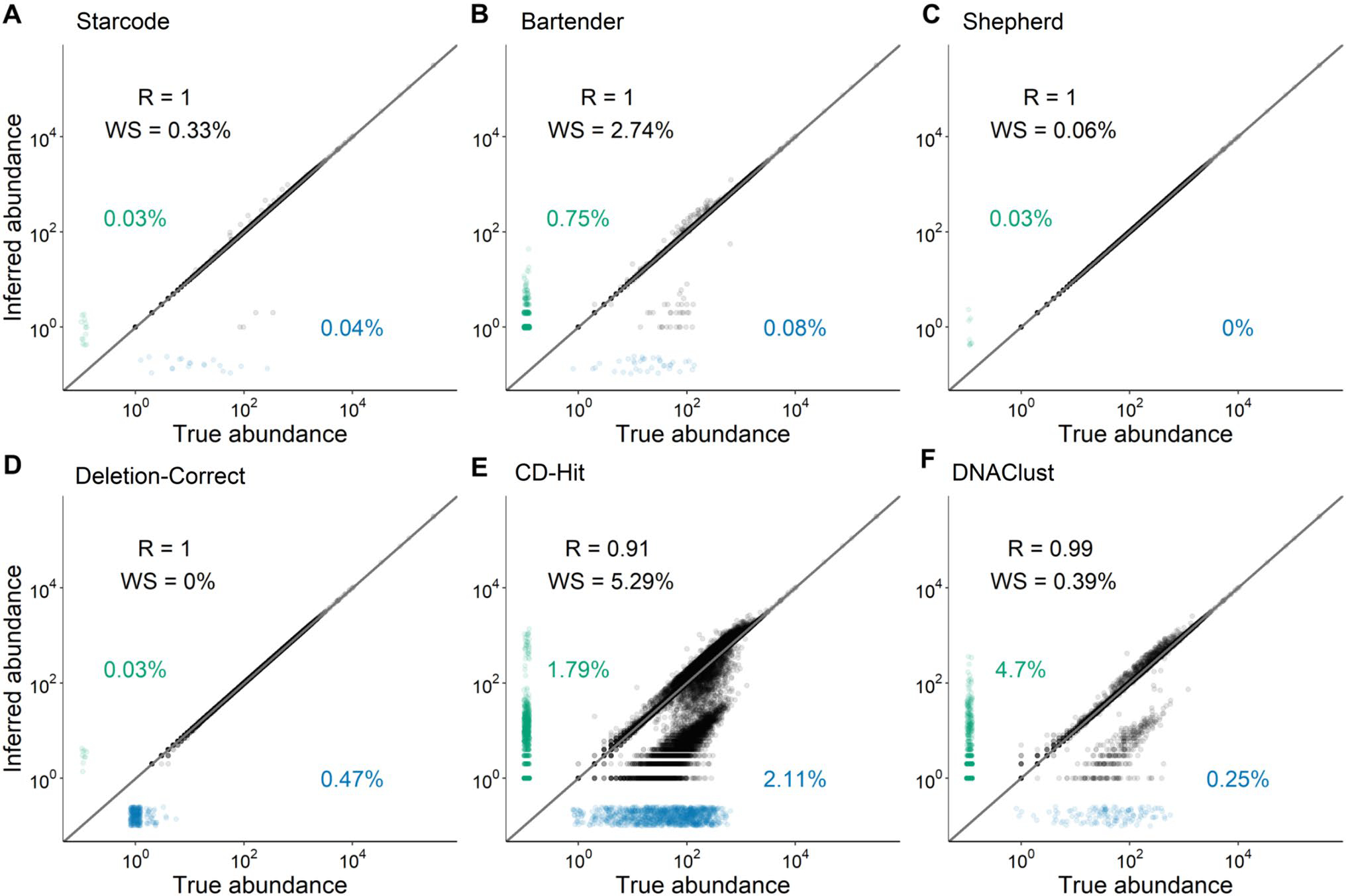
Comparison of error-correction methods. We tested six published error-correction methods on a simulated barcode dataset (see “Identifying barcodes in sequencing data” section and Methods for details). The true abundance of each barcode (x-axis) is shown against the inferred abundance of the barcode most closely associated with it after error correction (y-axis). “R” is the Pearson correlation coefficient of log-transformed data for the successfully inferred barcodes. “WS” is the fraction of barcodes where a wrong sequence was inferred by the error-correction method (see text). Blue points along the x-axis show false negatives, i.e., true barcodes that were not identified (numbers show percentages). Green points along the y-axis show false positives, i.e., identified barcodes that are not associated with a true barcode (numbers show percentages). The gray line is the diagonal *y* = x

We quantified three types of errors that occur during error correction. The first type of error, false negatives (indicated by blue points in Fig. 4), represents cases in which a true barcode is not included in the post-error-correction dataset, either because that barcode was error corrected to another true barcode or because it was excluded due to low read counts. For example, a read count threshold is responsible for most of false negatives for Deletion-Correct. The second is false positives, indicated by the green points in Fig. 4 which occur when an error barcode is not corrected and is instead identified as a true barcode. The third is “wrong sequence” errors, indicated by the “WS” numbers in Fig. 4E, which occur when the method correctly clusters error sequences with the parent sequence but incorrectly infers the parent sequence. Wrong-sequence errors are less costly for downstream analysis than false positives because they do not distort barcode frequencies, whereas false positives do. False positives are especially of concern in evolutionary lineage tracking experiments (e.g., Levy et al. 2015; Blundell et al. 2019; Venkataram et al. 2022), where such errors could cause one lineage with a beneficial mutation to appear as multiple, leading to errors in the distribution of fitness effects.

We found that all four barcode-specific methods successfully identified the vast majority of barcode sequences and correctly inferred lineage abundances (Pearson *R* = 1.0, Fig. 4A–D), while both generic methods performed poorly (Fig. 4E, F). Among the bespoke methods, Bartender has the highest WS and false-positive rates. One source of false positives is barcodes with indel errors, which Bartender does not attempt to correct. Starcode, Deletion-Correct, and Shepherd do attempt to correct them, although Shepherd does so only for single-base indels.

We next applied the barcode-specific methods on three empirical datasets (Levy et al. 2015; Johnson et al. 2019; Borchert et al. 2022) after having extracted barcodes using the alignment-based method. We found that Shepherd failed to identify many putative barcodes in these empirical datasets (Table S3). Specifically, the Levy, Johnson, and Borchert datasets contain 21,000, 10,000, and 2800 barcodes with at least 10 reads each, respectively, which are found by Bartender, Starcode, and Deletion-Correct but not by Shepherd. All lineages missed by Shepherd but identified by other methods have abnormal length, suggesting that Shepherd’s filtering criteria are too strict (see above). While Starcode consistently ran faster than the other methods, all methods took less than four minutes to run on a personal desktop computer, with the exception of Shepherd on the Levy et al. dataset, which took about 30 min. For all practical purposes, these execution times are sufficiently short to not substantially influence the choice of method.

In summary, we strongly recommend using barcode-specific methods for error correction, including Shepherd, Starcode, Bartender, and Deletion-Correct. It may be useful to use multiple methods in conjunction to better account for false positives, false negatives, wrong-sequence errors, and barcodes of abnormal length.

## Summary

We have reviewed the choices faced by researchers during the design, sequencing, and identification of random barcodes, as well as some of the implications of these choices for the quality of the data. Here, we provide a succinct summary of our main points.

### Design, Synthesis, and Integration

- The base composition of the barcode sequence strongly affects the error rates during sequencing-library preparation and/or sequencing process itself. In particular, long homopolymer or dinucleotide runs and extremely high or low GC content should be avoided.
- Barcode length and base composition limit the number of lineages that can be tracked. For barcodes with length 20 to 40 bp, the library size should be small enough that all but a small fraction of barcodes are at Hamming distance of at least four from each other.
- Barcode oligonucleotides synthesized with HPLC or PAGE purification and hand-mixed random bases result in barcode sequences with lower error rates.
- When choosing the integration locus, consider (i) its stability with respect to recombination events that can lead to barcode loss and (ii) the implications of genetic manipulations at the locus for the organism’s physiology.

### Sequencing

- Inline indices greatly expand multiplexing capacity and allow for detection of errors that arise due to template switching, index hopping, and primer cross-contamination.
- UMIs help detect whether noise in the data comes from a low number of template molecules, but their power to correct PCR biases is low.
- Read offsets help improve sequencing quality.
- Use of high-fidelity polymerase during PCR reduces amplification errors.

### Identification

- Regex and alignment approaches are both excellent at barcode extraction. Regex is faster, and alignment is slightly better at identifying abnormal barcode sequences.
- Error correction methods designed specifically for barcode data work much better than generic methods. Among the former, Shepherd is most accurate on simulated data but fails to recover barcodes of abnormal length, which appear in real data at non-negligible frequencies.

## Methods

### Measuring Variation in Synthesized Barcode Length

To measure variation in synthesized barcode length in the empirical datasets (Fig. 2E), we extracted barcodes using regular expressions that strictly match the 10 base pairs before and/or after the barcode sequence, with no length criteria for the sequence in between (see “Comparison of Barcode Extraction Methods” section). We then measured the percentage of barcodes with each possible length, ranging from 10 bp less than expected to 10 bp more than expected. We only considered barcodes with at least 20 read counts for this analysis to minimize the impact of amplification and sequencing errors on the distributions (see below).

### Estimation of Errors in Barcodes with Repetitive Sequences

To estimate the frequency of errors in repetitive barcode sequences (Figs. 2A, C), we extracted the barcode sequences from reads using the alignment method (see “Comparison of Barcode Extraction Methods” section). For both single nucleotides and every nucleotide pair (“dinucleotide”), we looked for barcodes with *N* repeats of that nucleotide or dinucleotide, with *N* ranging from 3 to 13. For the top 50 most abundant barcodes with a particular length run (excluding barcodes with less than or equal to 100 reads), we searched for putative error barcodes, which we require to have fewer reads than the true barcode, in which the number of repeats was increased or decreased by 1 or 2, but the rest of the barcode was identical. In parallel, we searched for single-nucleotide errors derived from each of these barcodes. We added the read counts from both the indel and single-nucleotide errors to each “true” barcode’s read counts in order to ensure an accurate denominator when calculating error rates. We report the total indel error rate in Fig. 2, which we calculate as the combined frequency of all four types of errors (insertions and deletions of one or two repeats).

### Simulating Barcode Designs and Measuring Barcode Statistics

We assessed some barcode design features (Fig. 2B, D, F and Figure S3) for four previously proposed designs and our new designs as follows. For each design, we simulated 100,000 random barcodes. We then measured the statistics of these simulated sets of barcodes, along with the corresponding empirical sets. For each empirical dataset, we used the list of barcodes derived from alignment-based extraction (see “Comparison of Barcode Extraction Methods” section), excluding any barcodes that are not the expected length. For each barcode, we measured the percentage of GC bases, the longest homopolymer run, and the longest dinucleotide run (Figs. 2B, D, F).

### Conditions for Preserving Barcode Diversity During Transformation

Assuming that $N=2^{I}$ barcodes are present at frequencies $\;f_{1},f_{2},\ldots ,f_{N}$ in the barcode pool prior to transformation and that K cells receive a barcode, the number of cells that receive each barcode is described by the multinomial distribution, so that the expected number of cells that receive barcode i is $Kf_{i}$. Therefore, the barcode diversity after transformation is high if a typical barcode is present in at most one cell, which happens when $Kf_{max}\ll 1$, where $f_{max}$ is the frequency of the most frequent barcode in the pool.

Assuming that barcode frequencies are distributed exponentially, we can estimate $f_{max}$ as follows. First, note that if all $f_{i}$ are drawn from an exponential distribution with mean μ, the fact that all $f_{i}$ must sum to 1 implies that $\mu =N^{-1}=2^{-I}$. Then, when N is large, the extreme value theorem states that $f_{max}/\mu -lnN$ is distributed according to the standard Gumbel distribution, whose expected value is γ≈0.577, the Euler-Mascheroni constant. Thus, in expectation, $f_{max}=2^{-I}(\gamma +I\;ln2)$.

### Distribution of Hamming Distances Between Barcodes

We generated barcode libraries with 10^2^, 10^3^, 10^4^, 10^5^, 10^6^, and 10^7^ fully degenerate barcodes of length 15, 20, or 30 bp. To reduce computation time, we utilized an approximate-nearest-neighbor algorithm as provided by the python Annoy library to find the nearest neighbor for every sequence in the dataset, which requires binary input. We, thus, encoded each position in the barcode using three bits (“A” = 000, “T” = 011, “C” = 101 and “G” = 110) so that every possible single-nucleotide substitution could be encoded by a change in two bits. This encoding, thus, ensures that nearest neighbor sequences in the binary encoding are also nearest neighbors in nucleotide space. We report the fraction of sequences with a Hamming distance to their nearest neighbor less than or equal to 0, 1, 2, 3, or 4 bp, averaged over five replicate simulations for each parameter combination.

### Identification of UMI Duplicates and Detection of Chimeric Reads

We report rates of chimeric reads and UMI duplicate based on the lineage tracking data from Venkataram et al. (2022). In that study, BarcodeCounter2 was used to extract barcodes from lineage tracking data. This software uses inline and Illumina index information to identify chimeric reads during sample demultiplexing and provides a count of UMI duplicates found for each barcode within each sequenced sample.

### Simulations of Bias Detection Using UMIs

To assess the utility of UMIs for correcting PCR biases (Figure S4), we carried out the following simulations. We start with a focal barcode whose frequency among template molecules is either 0.05 or 0.25. We vary the number of template molecules tagged with UMIs from 100,000 and 10 million, which spans the two regimes discussed in the main text. For each frequency and number of template molecules, we generate the “post-library-preparation pool” by (i) associating every template molecule with a unique UMI and (ii) multiplying the initial abundance of the focal barcode by the bias factor indicated in Figure S4. We then randomly sample 1 million reads from the post-library-preparation pool. We remove the UMI duplicates and compare the resulting frequency of the focal barcode with its true frequency.

### Comparison of Barcode Extraction Methods

We implemented custom regular expression and alignment software to extract barcodes from each of six datasets. To extract barcodes by regular expressions, a set of five custom regular expressions of increasing leniency were composed for each dataset to extract barcode sequences based on the read sequences from each dataset. For example, we used these five regular expressions successively to find barcodes in the reads from the Borchert et al. (2022) dataset (stopping if the regular expression found a match):


\D*?(CGTA CG)( \D{20})(AGAG AC) \D* (exact match to barcode sequence)


\D*?(CGTA CG)( \D{19,21})(AGAG AC) \D* (allows single base indels)

\D*?(CGTA CG)( \D{18,22})(AGAG AC) \D* (allows two-base indels)

\D*?(CGTA CG){ e<=1}(\D{20})(AGAG AC){ e<=1}\D*

(allows one error in the flanking sequences)

\D*?(CGT ACG ){e<=1}(\D{18,22})(AGA GAC )

{e<=1}\D* (allows one error in the flanking sequences and two-base indels)

To extract barcodes by alignment, we used BLASTn + v 2.6.0 (Altschul et al. 1990; Camacho et al. 2009) to identify the location of the constant sequences flanking each barcode within the read, and used these positions to extract the barcode sequence. BLASTn + was run with the parameters -word_size 6 -outfmt 6 -evalue 1E0 -maxhsps 1. The word_size and evalue parameters were selected so that matches could be found even for very short sequences, as the default parameters are typically unable to find matches even if the exact target sequence is present in the read. However, different parameter choices may be necessary for different datasets. Other read mapping software (e.g., Bowtie2 or BWA) can also be used to align entire reads to a template for barcode extraction (Jasinska et al. 2020).

### Comparison of Error-Correction Methods

#### Simulations of Barcode Data with Errors

To simulate barcode data with a range of frequencies including high-frequency outliers, we first drew 99,895 barcode abundances from an exponential distribution with mean 1, 100 barcode abundances from an exponential distribution with mean 10, and 5 barcode abundances from an exponential distribution with a mean of 1000. We assigned each abundance to a randomly generated 20-bp barcode (“N20”). We then drew a number of reads associated with each barcode from a Poisson distribution with a mean of the frequency of the barcode multiplied by 25 million (such that we expect a total of approximately 25 million reads). For any barcode with a mononucleotide run of 5 or more base pairs, we first simulated indel errors, using our empirical data on the rates of these events (Fig. 2) to draw a Poisson-distributed number of reads with a single-base insertion or deletion. This indel simulation process was carried out recursively such that multiple-base indels were possible. Next, we simulated single nucleotide errors for each read at a rate of 0.4% per base. The final simulated dataset consists of a single row for each unique barcode that was “read” in this process, associated with a number of reads and the “true” barcode from which it is derived.

#### Comparison of Error-Correction Methods

We tested six error-correction methods (Bartender v1.1.0, DNAClust v3, Starcode v1.4, Shepherd downloaded Aug 15 2022, CD-Hit v4.8.1 and Deletion-Correct, provided in this manuscript) on each of four datasets (Levy, Borchert, Johnson, and the simulated dataset). Each program was run with the following parameters, where *L* is the length of the barcode, including anchor sequences.


Starcode ‘-d 3 -s’


Bartender ‘-d 3’

Shepherd ‘-l L -bft 4 -eps 3’

Deletion-Correct: min_counts_for_centroid=2, max_edits=3, poisson_error_rate=0.1

CD-Hit ‘-c {1–3.1/L} -n 6’

DNAClust ‘-s {1–3.1/L} -k 6’

While a complete analysis of the parameter space for each of these programs is beyond the scope of this paper, we encourage researchers to consider these choices carefully in the context of their data. In particular, edit distance thresholds can alter the risk of failing to correct error barcodes. Based on the previously published error rates of PCR and Illumina Sequencing of ~ 0.5%, we expect an edit distance threshold of 3 to correctly cluster 99.9% of error sequences in the datasets we analyze here, while also correctly distinguishing most of the distinct barcode sequences (Fig. 4). We, thus, used this edit distance threshold for most analyses. The K-mer parameters used by DNAClust (−k) and CD-Hit (−n) only affect the speed of the computation and not the accuracy, so we did not explore the impact of these parameters. We used the default log Bayes factor parameter for Shephard (−bft) as recommended by the authors of the software for discriminating real and error sequences.

Programs were run on a personal desktop computer with an AMD Ryzen5 1600 3.2 GHz processor and 16 GB of RAM. Software with multithreading support was run with 10 threads / allocated processing cores and 5000 MB of allocated memory.

## Supplementary Material

Supplementary Information

## Data Availability

Simulated read data and all code used for simulations, analysis, and generating figures have been deposited on Zenodo at https://doi.org/10.5281/zenodo.7052124.

## Appendix

### Genotyping Clones at a Barcode Locus

A common task when using barcoded strain libraries is to identify the barcodes for individual clones isolated from the library. The traditional approach, based on Sanger sequencing, is effective for a small number of clones, but it becomes prohibitively expensive and labor intensive at ∼10^2^ clones. At larger scales, approaches that leverage next-generation sequencing technologies are preferred.

The most straightforward cheaper alternative to Sanger sequencing is to individually amplify the barcode of each clone, tag it with a unique combination of indices and sequence it on the Illumina platform. Since this approach involves the same number of DNA extractions and PCR reactions as the Sanger approach, the cost of this approach scales linearly with the number of samples. The savings come from the reduction of sequencing costs per sample: sequencing of a sample with the Sanger technology currently costs about 2 USD, while the cost is less than 0.02 USD per sample on the Illumina MiSeq platform when sequencing 10,000 clones.

An even cheaper alternative for genotyping many clones is a pooled sequencing strategy sometimes referred to as “Cartesian pooling,” “Compressed sensing,” or the “Sudoku method” (Barillot et al. 1991; Erlich et al. 2009; Shental et al. 2010; Vandewalle et al. 2015; Baym et al. 2016). The idea is to pool clones into multiple groups, such that each clone is present in several groups, prepare one Illumina library per group, sequence them, and then infer the genotypes of all clones based on the knowledge of their presence/absence in each group. For example, clones can be arrayed into a 3-dimensional grid of p plates, each with r rows and c columns, e.g., in a series of 96-well plates. This would result in p+r+c groups, each containing all clones in a given plate, row or column across the entire collection. In this arrangement, each clone is present in only one specific combination of plate, row and column groups, and no two clones are present in the same combination of groups. In other words, group combination serves as a clone’s unique fingerprint. Further, if all clones have distinct barcodes, there will be only one barcode sequence present in any given combination of plate, row and column groups. In other words, each sequence will have a unique fingerprint, through which it can be assigned to the correct clone. While this strategy requires some additional work pooling clones into groups, the overall cost scales approximately as $K^{1/3}$, where K is the number of clones, since only about $K^{1/3}$ DNA extractions and PCR reactions are required. For example, a library of 960 clones can be characterized using 30 pools (10 plate pools, 8 row pools and 12 column pools). The efficiency can be further improved by using additional “dimensions” for pooling and ensuring that all groups have similar numbers of clones (Barillot et al. 1991).

A key limitation of the Cartesian pooling approach occurs when multiple clones have the same barcode. In this case, some sequences are present in more than one group combination (i.e., they have multiple fingerprints) which makes the association of sequences with clones non-unique. For example, consider a collection of 96 clones, pooled by row and column, where clones present in wells A5 and D7 have the same barcode. In this scenario, row groups A and D as well as column groups 5 and 7 will have this particular barcode sequence. Thus, the barcode could be assigned to any of four wells: A5, A7, D5, and D7. Resolving these degeneracies may require additional genotyping (Barillot et al. 1991).
