# Supplementary material for: Best Practices in Designing, Sequencing, and Identifying Random DNA Barcodes
Source: J Mol Evol. Author manuscript; Available in PMC 2023 Jul 10. (PMC10276077; doi:10.1007/s00239-022-10083-z)
Supplement: Supplementary Information [file NIHMS1899469-supplement-Supplementary_Information.pdf]

Supplemental Figures

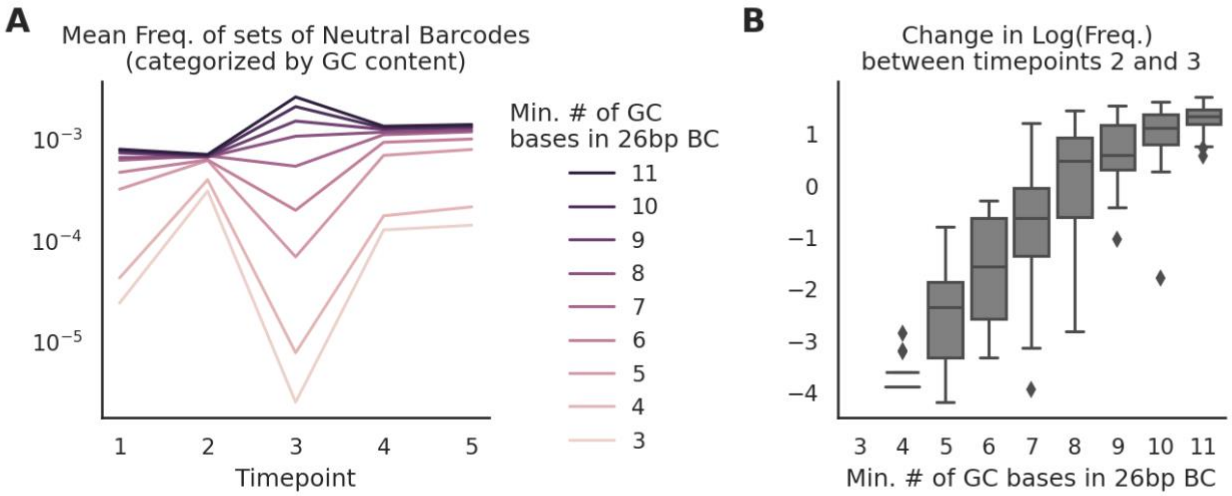

Figure S1. Evidence that GC content affects barcode frequencies. (A) Dynamics of the mean frequency of putatively neutral lineages carrying barcodes with different GC content (unpublished data). This experiment featured two adjacent 26 bp barcode loci. GC content is measured as the minimum number of G or C bases in whichever of the two barcode loci has fewer G or C bases. Different lines show the mean frequency during a single fitness assay of sets of lineages with the specified GC content. In the absence of GC-content-dependent biases, all lines should be parallel. Barcode frequencies also generally should not correlate with GC content, and we observed no such correlation in repeated sequencing of this library. The fact that the GC-content bias is highly variable between timepoints suggests that subtle uncontrolled variation in library preparation conditions can have a large effect on the degree of this bias. **(B)** Variation in the change in log-frequency between timepoints 2 and 3 shown in **(A)** within and between barcodes stratified by GC content. This change is expected to be independent of GC content. We note that this is the strongest example of bias we have observed so far.

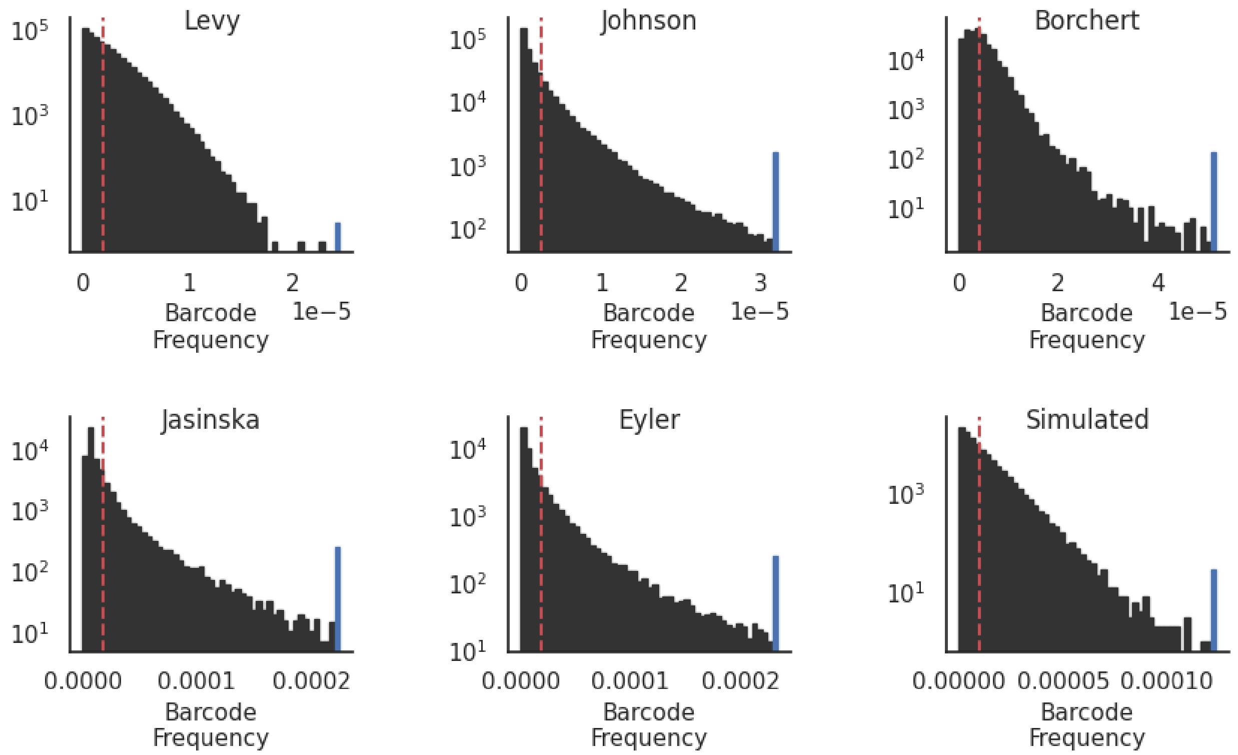

Figure S2. Barcode frequency distributions at time zero. Each panel shows the distribution of barcode frequencies at time zero after error-correction using Deletion-Correct for the five indicated datasets and for a simulated dataset (see Methods for details). x-axis is scaled to the expected barcode frequency if all barcodes were at uniform abundance (red dashed line). The data is clipped, such that the blue bar in each graph represents all barcodes with frequencies at least 12 times higher than the expected uniform frequency.

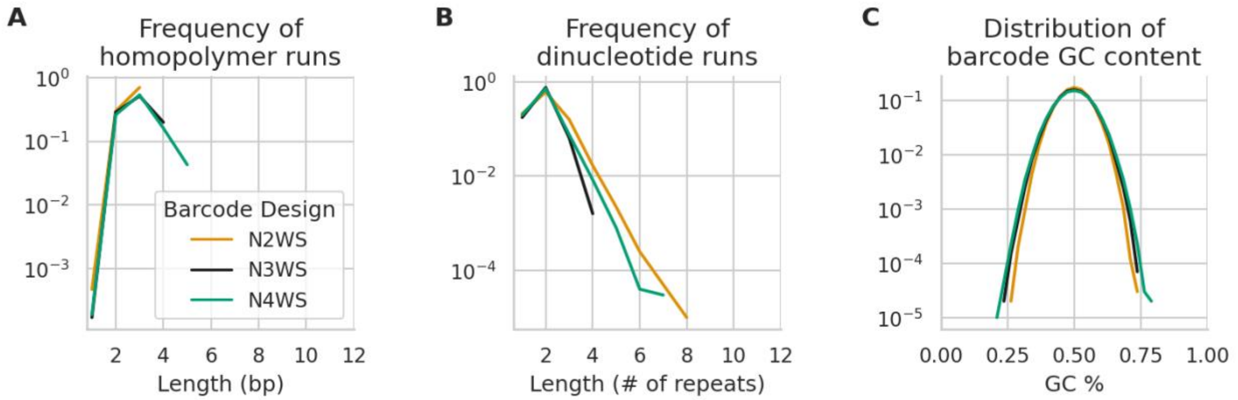

Figure S3. Barcode design features and error rates for three new designs. This figure shows the same statistics as in Figure 2B,D,F, but for three new barcode designs with either 2 (N2WS), 3 (N3WS), or 4 (N4WS) fully degenerate nucleotides between each "WS" anchor. All three designs have a total length of 38 bp. **(A)** The frequency of homopolymer runs of different length, analogous to Figure 2B. **(B)** The frequency of dinucleotide runs of different length, analogous to Figure 2D. **(C)** The distribution of GC content in barcodes, analogous to Figure 2F.

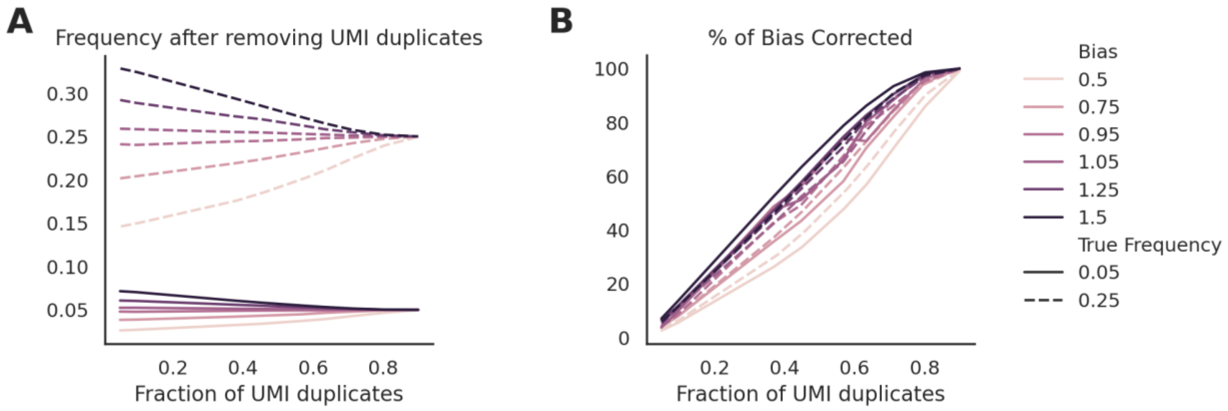

Figure S4. Removal of UMI duplicates partially corrects amplification biases. (A) The frequency of a simulated focal barcode with a library-preparation bias (e.g. PCR amplification bias) after removing UMI duplicates as a function of the fraction of UMI duplicates. **(B)** The percent of the difference between the true frequency and read-based frequency of the focal barcode that is corrected as a function of the fraction of UMI duplicates. Data is the same as in **(A)**.

Supplemental Tables

Study	Read file (SRR accession)	Description	Approx. library size (K)	Reads	Barcode sequence	Length / information
**Johnson et al. 2019**	SRR9850741	RB-TnSeq (*Saccharomyces cerevisiae*), time zero	400,000	18,560,760	NNNNCANNNNCANNNNCANNNNCANNNN	28 bp / 40 bits
**Levy et al. 2015**	SRR5747458	Lineage tracking (*Saccharomyces cerevisiae*), time zero	500,000	142,918,126	NNNNNAANNNNNAANNNNNTTNNNNN	26 bp / 40 bits
**Jasinska et al. 2020**	SRR10556795	Lineage tracking (*Escherichia coli*), initial library	50,000	6,131,498	NNNNNNNNNNNNNNN	15 bp / 30 bits
**Eyler et al. 2020**	SRR10704145	Cell-line lineage tracking (glioblastoma), time zero	50,000	7,465,619	WSWSWSWSWSWSWSWSWSWSWSWSWSWSWS	30 bp / 30 bits
**Ge et al. 2020**	SRR9162708	Cell line lineage tracking (breast cancer), JQ1 treatment, passage 11, replicate 3	80,000	11,809,554	WSWSWSWSWSWSWSWSWSWSWSWSWSWSWS	30 bp / 30 bits
**Borchert et al. 2022**	SRR18112661	RB-TnSeq (*Pseudomonas putida*), M9+20 mM D-glucose, replicate A, time zero	200,000	5,618,453	NNNNNNNNNNNNNNNNNNNN	20 bp / 40 bits

Table S1. Datasets reanalyzed in this paper. Approximate library sizes are estimated after error correction using Deletion-Correct.

Dataset	No barcode extracted by either method	At least one barcode extraction succeeded				
		Barcodes match	Match with 1–3 edits	Mismatch	Regex failed	Alignment failed
**Johnson et al. 2019**	1.67%	98.058% (66,266 BCs)	0.589% (409 BCs)	0.018% (11 BCs)	1.300% (973 BCs)	0.036% (8 BCs)
**Levy et al. 2015**	3.82%	98.726% (80,386 BCs)	0.155% (141 BCs)	0.057% (49 BCs)	0.995% (833 BCs)	0.067% (63 BCs)
**Jasinska et al. 2020**	1.87%	99.302% (33,555 BCs)	0.269% (81 BCs)	0.015% (6 BCs)	0.413% (127 BCs)	0.001% (1 BCs)
**Eyler et al. 2020**	2.87%	97.588% (36,754 BCs)	0.310% (238 BCs)	0.045% (39 BCs)	2.056% (1618 BCs)	0.001% (1 BCs)
**Ge et al. 2020**	3.33%	98.837% (16,492 BCs)	0.095% (45 BCs)	0.080% (53 BCs)	0.981% (480 BCs)	0.007% (6 BCs)
**Borchert et al. 2022**	5.94%	98.468% (66,554 BCs)	0.165% (132 BCs)	0.416% (385 BCs)	0.314% (268 BCs)	0.638% (596 BCs)

Table S2. Comparison of two barcode extraction methods on 6 published datasets. Each row represents one barcode sequencing dataset used for testing. The first 100,000 reads were used to test a regex-based barcode extraction method and an alignment-based barcode extraction method (see Methods). We report the percentages of reads and number of unique barcodes identified by both methods or only one method (e.g. “Regex failed” indicates cases where the alignment method identified a barcode in the read but the regex method did not).

Dataset	Number of extracted sequences	Starcode	Bartender	Shepherd	Deletion-Correct
**Johnson et al. 2019**	719,584	447,068	455,360	426,998	381,047
**Levy et al. 2015**	2,086,173	500,565	539,250	480,067	500,806
**Borchert et al. 2022**	336,219	260,684	266,068	246,583	236,428
**Simulated**	1,544,849	99,581	100,257	99,615	99,152

Table S3. Number of identified barcodes before and after error correction for three empirical datasets and simulated data across four error correction methods.
